# Using machine learning to define mepolizumab treatment response at 2 years in patients with chronic rhinosinusitis with nasal polyps

**DOI:** 10.3389/falgy.2026.1710163

**Published:** 2026-02-13

**Authors:** María Sandra Domínguez-Sosa, María Soledad Cabrera-Ramírez, Miriam del Carmen Marrero-Ramos, Carlos Cabrera-López, Teresa Carrillo-Díaz, Jesús Benítez-Rosario, Carmen Delia Dávila-Quintana

**Affiliations:** 1 Hospital Universitario de Gran Canaria Dr Negrin, Las Palmas de Gran Canaria, Spain; 2 Universidad de Las Palmas de Gran Canaria, Las Palmas de Gran Canaria, Spain

**Keywords:** chronic rhinosinusitis, machine learning, mepolizumab, monoclonal antibody, nasal polyps, response

## Abstract

**Introduction:**

Using machine learning to identify clinical biomarkers for determining optimal response to mepolizumab in chronic rhinosinusitis with nasal polyps.

**Methods:**

Single center retrospective observational study with 84 CRSwNP patients treated with mepolizumab. We evaluated 4 machine learning algorithms: Decision Tree, Logistic Regression, K-Nearest Neighbors and Extreme Gradient Boosting. K-Fold cross-validation incorporating hyperparameter optimization in the process was used to ensure robustness and prevent overfitting.

**Results:**

After 6, 12 and 24 months, SNOT-22, VAS overall symptom score, VAS-smell, asthma control test (ACT) and nasal polyp score (NPS) significantly improved (*p* < 0.001). 44.1% of patients were classified as “super-responders” after 2-year of Mepolizumab treatment based on EPOS/Euforea criteria. XGBoost emerged as the most accurate for predicting super-response to mepolizumab, achieving an ROC- AUC of 0.766. In contrast, Logistic Regression was the least effective for predicting sustained super-response at 24 months, with an ROC-AUC of 0.628. Significant predictors included Blood Neutrophilia and Blood Eosinophilia where higher baseline scores were linked to higher probabilities of super-response at 24 months. Shapley Additive Explanations were employed to identify the most critical baseline features and to visualize their directional impacts on treatment responses.

**Conclusions:**

Machine learning models, particularly XGBoost, can predict real-world super-response to mepolizumab in severe CRSwNP by identifying key predictors like high baseline BEC, high baseline BNC and AERD comorbidity. These insights have the potential to refine CRSwNP treatment strategies and support clinical decision-making, ultimately enhancing patient outcomes by predicting treatment response prior to starting medication

## 1 Introduction

Chronic rhinosinusitis with nasal polyps (CRSwNP) is a phenotype of chronic rhinosinusitis (CRS) characterized by systemic eosinophilic type 2 inflammation in which interleukin (IL)-5 plays a key pathogenic role (1).

Previous studies have indicated that up to 60% of CRSwNP patients have concomitant asthma (2). Patients with comorbid asthma have significantly greater sinus inflammation and are more likely to undergo sinus surgery (2, 3). Asthma and CRSwNP share pathophysiologic mechanisms in which IL-5 and eosinophil-driven inflammation play an important role (4).

According to Stevens WW et al. (5, 6), the term aspirin-exacerbated respiratory disease (AERD), also called Non-steroidal Anti-inflammatory Drug-exacerbated respiratory disease (NSAID-ERD), or Samter's triad refers to patients with CRSwNP and asthma who also develop respiratory reactions to COX-1 inhibitors. Patients with AERD are more likely to have recurrence of CRSwNP and require revision endoscopic sinus surgery (ESS) and treatment with systemic corticosteroids (SCS) for CRSwNP (5).

Biologic therapies that target type 2 inflammation are available for the treatment of refractory CRSwNP associated with severe disease, asthma, AERD and recurrence following surgery (7, 8). The advent of biologic therapies, including anti-IL-5 agents such as mepolizumab, has significantly improved disease control in selected patient populations (7).

Mepolizumab is a humanized IgG1/k class monoclonal antibody (mAb) which selectively binds to interleukin-5 (IL-5), a cytokine that promotes eosinophil development and survival (9).

However, despite targeted biologic treatments, a significant proportion of CRSwNP patients are non-responders (10). Non-responder rates of 25% were observed in SYNAPSE phase 3 trials (11). CRSwNP treatment is challenging because each phenotype needs a different approach. Responses are variable, underscoring the need for reliable predictive tools to guide therapeutic decisions (12).

Machine learning (ML) can help identify patterns and strategies to develop more accurate treatments (13). In this study, we apply four machine learning models—Decision Tree (DT), Logistic Regression (LR), k-Nearest Neighbors (KNN), and Extreme Gradient Boosting (XGBoost)—to strengthen patient classification and biologic-response prediction in CRSwNP. Unlike conventional statistical comparisons that primarily test associations, ML learns complex non-linear interactions and higher-order feature relationships, revealing subtle response phenotypes in heterogeneous cohorts (14, 15). Because clinical datasets often have limited sample sizes and class imbalance, we use hyperparameter optimization with stratified k-fold cross-validation to obtain more trustworthy, less biased estimates of out-of-sample performance and to reduce overfitting (16). DT provides interpretable rule-based splits, LR offers transparent linear decision boundaries for classification, KNN exploits local similarity among instances, and XGBoost's regularized boosting improves accuracy while controlling model complexity. Together, these complementary models provide predictive insight beyond traditional methods for assessing biologic response in CRSwNP (14). Peripheral blood inflammatory markers, such as blood eosinophilia, total IgE, and blood neutrophilia, have emerged as clinically relevant indicators of systemic inflammation and immune endotype. These biomarkers are increasingly being integrated into ML models to predict recurrence (17, 18), classify inflammatory endotypes (19–21), and support personalized treatment strategies (20).

This study aims to enhance precision medicine in CRSwNP by identifying patients who are most likely to respond favorably to mepolizumab using ML techniques. It also identifies the clinical variables that influence treatment outcomes.

As a collateral secondary objective, this study also evaluated the sustained therapeutic efficacy and safety of mepolizumab over a 2-year period and across multiple follow-up episodes in a real-world cohort of CRSwNP and asthma patients.

## 2 Materials and methods

### 2.1 Study design

A retrospective single-center observational study in a real-life setting was conducted to review clinical data from adult patients with CRSwNP and comorbid asthma attending the multidisciplinary airway diseases unit of our health area. Patients started 100 mg mepolizumab treatment, administered subcutaneously once every 4 weeks, in addition to the standard of care from January 2018 until December 2024. Some of the patients were previously included in an earlier publication from the same authors (7).

The study was approved by the Hospital Ethics Committee (CEIm code: 2024-588-1), and all patients provided written informed consent before the collection of data.

CRswNP was diagnosed in accordance with the European Position Paper on Rhinosinusitis and Nasal Polyps (EPOS) 2020 criteria (22). Asthma was diagnosed in accordance with the definition of the 2022 Global Initiative for Asthma (23), and allergic rhinitis according to the 2019 Allergic Rhinitis and its Impact on Asthma (24). All patients with severe uncontrolled asthma met ATS/ERS criteria (25).

Since mepolizumab was not approved for CRSwNP until December 2021, the decision to initiate mepolizumab therapy before this date was taken individually by pneumologists and allergists in our health airway unit, considering asthma comorbidity characteristics.

The pre-treatment demographic characteristics and medical histories of 84 patients were reviewed and analyzed, including smoking history, endoscopic sinonasal surgery (ESS), comorbid asthma, aspirin-exacerbated respiratory disease (AERD), allergic rhinitis, and atopy.

We included patients older than 18 years with symptoms of severe CRSwNP, defined as follows: endoscopic nasal polyp score (NPS) ≥ 4, with a minimum score of 2 in each nasal cavity; Sinonasal Outcome Test score (SNOT-22) ≥ 40; ≥1 ESS in the last 10 years; inadequate symptom control with standard of care and/or failure of previous ESS and, exceptionally, CRSwNP and asthma patients with a medical contraindication to sinonasal surgery. Exclusion criteria included asthma exacerbations requiring hospitalization within 4 weeks prior to inclusion, immunosuppressive therapy before inclusion, and long-term corticosteroid therapy for chronic autoimmune diseases.

The following outcomes were assessed at baseline and after 6, 12, and 24 months of treatment: validated SNOT-22 score, evaluation of clinical sinonasal symptoms (nasal obstruction, nasal discharge, olfactory disorders, facial pain and overall symptom scores) using a visual analogue scale (VAS) symptom score; nasal polyp score (NPS: 0–4) expressing the endoscopic extension of nasal polyps. NPS was the sum of left and right nostril scores (0–8) (26). Hematology samples were collected from peripheral blood to determine total serum IgE and blood eosinophil counts (BEC) and blood neutrophil counts (BNC) at baseline and 6, 12, and 24 months.

*Atopic profile and aeroallergen sensitization* were determined with a positive skin prick test as described by the European Academy of Allergy and Clinical Immunology (27). We also measured prednisone intake. Sinonasal computed tomography (CT) scans were performed at baseline. These CT scans were scored according to the Lund-Mackay staging system (28).

*Asthma control* was assessed using the Asthma Control Test (ACT), a self-administered questionnaire. Asthma with an ACT score greater than 19 is considered well controlled (29). A change of 3 points is likely to indicate a clinically meaningful change in asthma control (30).

*Clinical AERD diagnosis* was confirmed with the symptom triad of asthma, CRSwNP and respiratory reactions to aspirin and other NSAIDs (5).

*Clinical quality of life improvement* was defined as an improvement of ≥8.9 points, corresponding to at least one minimal clinically important difference (MCID) in the SNOT-22 score (31).

*Response to biologics* was evaluated according to the adapted EPOS/EUFOREA 2023 update criteria (32) and the quantitative thresholds proposed by the EUFOREA expert board in 2021 (33). We divided patients based on treatment clinical response as follows: (a) NPS reduction (at least 1 point); (b) SNOT-22 score reduction (at least 8.9 points); (c) improved VAS-smell score (at least 3 points); (d) reduced impact of comorbidities (ACT score reduction at least 3 points); and (e) no need for rescue treatment (no SCS intake or need for surgery). Based on the above criteria, patients were divided into 4 groups: super-responder (5 criteria); good-excellent responder (4 criteria); poor-moderate responder (1–3 criteria); non-responder (0 criteria).

Adverse events associated with the use of mepolizumab during the observation period were collected. II.

### 2.2 Statistical analysis

Patient characteristics were summarized using descriptive statistics (Table 1). Categorical variables are expressed as frequencies (*n*) and percentages (%), while continuous variables are presented as median and interquartile range (IQR, 25th–75th percentile) due to a non-normal distribution as determined by the Shapiro–Wilk test. Changes in scores over time were statistically assessed using the Wilcoxon signed-rank test and Hodges-Lehman estimator (Table 2). The Mann–Whitney *U-*test was used to assess whether the outcome scores at 2 years were significantly different between the 2 groups: super-responders and non-super-responders. Chi-square or Fisher's exact tests were used for comparing categorical variables.

**Table 1:** Patient characteristics at baseline (*n* = 84).

Personal characteristics	Median (IQR)	*n* (%)
Age (years)	57 (45–63)	
Gender		
Male		32 (38.1%)
Female		52 (61.9%)
Current smoker, *n* (%)		10 (11.9%)
Comorbidities and allergies		
Asthma		84 (100%)
ACT score	17 (12–20)	
AERD		46 (54.8%)
Baseline biomarkers		
Blood eosinophils/μL	325 (117–500)	
Blood eosinophils/μL (≥150)		60 (71.4%)
Blood neutrophils/μL	3,920 (3,140–5,180)	
Serum total IgE IU/mL	104 (28–179.1)	
Serum total IgE IU/mL (≥100)		46 (54.8%)
FeNO ppb	36.5 (22.5–48.8)	
FeNO ppb (≥35)		50 (59.5%)
Control of disease		
Endoscopic sinus surgery		74 (88.1%)
Post-biologic surgeries		5 (6%)
Previous biologic therapy		8 (9.5%)
Corticosteroid dependence		77 (91.7%)
Scores		
NPS	4 (3–6)	
Lund-Mackay CT staging score	20 (16.5–22)	
SNOT-22 total score	68 (54–84.8)	
VAS-total	7 (6.3–8.1)	
VAS-smell	10 (10–10)	
VAS-nasal obstruction	10 (8–10)	
ACT	17 (12–20)	

Data are expressed as absolute and relative percentage frequency for qualitative data, median and interquartile range (IQR) for quantitative data. ACT, asthma control test; AERD, aspirin and nonsteroidal anti-inflammatory drug-exacerbated respiratory disease; FeNO ppb, fractional exhaled nitric oxide parts per billion; IgE, immunoglobulin E; NPS, nasal polyp score; SNOT-22, sinonasal outcome test 22; VAS, visual analogue scale.

**Table 2:** Baseline values and change scores in clinical parameters.

Variables	Baseline	Δ6 months vs. baseline^b^	Δ1 year vs. baseline^b^	Δ2 years vs. baseline^b^	Δ1 year vs. 2 years^b^
	(*n* = 84)	(*n* = 84)	(*n* = 70)	(*n* = 59)	(*n* = 59)
SNOT-22 total score	68 (54;84.8)^a^	−50 (−56;−45)***	−55 (−60.5;−49.5)***	−56.5 (−63.5;−50)***	−0.5 (−2;1)
VAS overall symptom score	7 (6.3;8.1)^a^	−2.2 (−2.6;−2)***	−3.5 (−4;−3.1)***	−5.1 (−5.7;−4.5)***	−1.1 (−1.4;−0.7)***
VAS-smell	10 (10;10)^a^	−2.5 (−3;−2)***	−3 (−4;−2.5)***	−5.5 (−6;−4)***	−1.5 (−2;−1)***
NPS	4 (3;6)^a^	−2.5 (−3;−2)***	−2 (−2.5;−2)***	−2.5 (−3;−2)***	0 (0;0.5)
ACT	17 (12;20)^a^	4 (3;5.5)***	5 (3.5;6)***	6.5 (5.5;8)***	1.5 (0.5;2)***

^a^ Median and IQR at baseline.

^b^ Hodges–Lehmann Estimator [95% Confidence Interval (CIs)]. The median differences between the scores of both periods and 95% CIs were estimated with the Hodges–Lehmann method. *P* value is from the Wilcoxon rank-sum test. Statistically significant difference at:.

*** *p* < 0.001.

Spearman correlation coefficients were calculated to determine the relationship between changes in outcome scores from baseline to 24 months and biomarkers.

Data preprocessing was performed using IBM SPSS Statistics, version 29.0. Statistical analyses and graph generation were conducted using Python (version 3.12.7) within a Jupyter Notebook environment. Statistical significance was determined at *p* < 0.05.

### 2.3 Machine learning modeling

#### 2.3.1 Machine learning model construction and evaluation

We evaluated various predictive machine learning models, including DT, LR, KNN, and XGBoost, to identify reliable predictors of mepolizumab super-response, based on adapted EPOS/EUFOREA 2023 criteria (32) in 59 samples after 2 years of treatment. The clinical variables included in the ML models—AERD status, baseline blood eosinophil and neutrophil counts, total IgE, and Lund-Mackay score—were selected based on their consistent use and reported predictive value in prior studies evaluating biologic efficacy in CRSwNP (1–4). These variables reflect key markers of inflammation and severity of type 2 disease, which are mechanistically linked to treatment response. Age, sex, and surgical history were considered but excluded because previous evidence suggests they have limited predictive impact compared to inflammatory and radiologic markers. To optimize model performance and external validity, we prioritized clinically relevant, biologically plausible predictors.

Performance metrics, such as sensitivity, specificity, F1, area under the receiver operating characteristic curve (ROC AUC), and overall accuracy, were calculated for each model to predict super-response. To ensure robust stratification and reliable evaluation, we used K-Fold cross-validation with 5 folds, averaging each model's evaluation metrics over the separate validation sets to obtain a robust estimate of generalization performance.

#### 2.3.2 Model interpretation

To improve the reliability and interpretability of our best model, we identified and ranked essential features using Shapley Additive Explanations (SHAP), which quantify each feature's impact on the model's predictions. We computed the mean absolute SHAP value [mean(|SHAP|)] for each feature across all patients. The SHAP value for a given patient and feature quantifies the contribution of that feature to the model's prediction for super-responder status, with higher mean(|SHAP|) values indicating greater overall influence (regardless of direction) on model output. This approach enables both global and individualized interpretation of feature impact within complex, non-linear machine learning models (15). Likewise, the potential non-monotonic effects of the variables were analyzed through partial dependence plots (PDPs) (34).

PDPs offer valuable interpretability in biomedical modeling by isolating the effect of individual features on predicted outcomes. By visualizing how changes in a single variable influence model prediction, they enhance transparency and support the clinical relevance of machine learning outputs.

The machine learning models were implemented using Scikit-Learn and XGBoost. Data handling and preprocessing were performed with Pandas and NumPy. Visualization tasks incorporated matplotlib, seaborn, plotly, dtreeviz, and PIL, while interpretability analyses employed SHAP and PDPbox.

## 3 Results

### 3.1 Patient characteristics at baseline and changes in parameters

A total of 84 consecutive patients were progressively enrolled in the study. No patients discontinued treatment or dropped out of the study and all participants completed the 6-month follow-up, 70 (83%) had completed 12-months of follow-up and 59 (70%) completed 24-months. Patients who joined the study between 2018 and 2024 were monitored, with data collected at baseline and at predefined intervals of 6, 12, and 24 months. The variation in the number of patients available at each follow up interval is solely attributable to the staggered initiation of treatment, and not to attrition or treatment discontinuation.

Patients’ baseline characteristics and type 2 inflammation biomarkers are shown in Table 1. Comorbidities like asthma and AERD were present in 100% and 54.8% of patients respectively.

Most patients (71.4%) presented blood eosinophil levels ≥150 cells/μL and 54.8% exceeded levels of 100 IU/mL. The median blood neutrophil count was 3,920 cells/μL (3,140–5,180).

Many patients, 74 patients (88.1%) had previously undergone ESS, and 5 (6%) required additional surgical interventions post-biologic therapy. A minority, 8 (9.5%), had received prior biologic omalizumab treatment for severe uncontrolled asthma**,** while 77 patients (91%) were receiving SCS at the time of starting mepolizumab. No treatment-associated adverse events were reported.

Table 2 presents the progression of clinical outcomes over time to evaluate the effectiveness of mepolizumab treatment. After 6, 12, and 24 months, all scores improved significantly (*p* < 0.001). We reported a significant reduction of SNOT-22 score, with a baseline median (IQR) value of 68 (54;84.8) and reductions in terms of median differences (IQR) of 56.5 (−63.5; −50) at 24 months. Baseline values for the VAS overall symptom score and VAS-smell score were initially 7 (6.3; 8.1) and 10 (10; 10), respectively. Sustained improvement was observed throughout the study period**.** A significant improvement in NPS and ACT score was also reported (*p* < 0.001), with a reduction of −2.5 (−3; −2) in NPS and an improvement of 6.5 (5.5; 8.5) in ACT score at 24 months, respectively.

### 3.2 Characterizing super-responders vs. non-super-responders

After 2 years of follow-up treatment, 44.1% (26/59) of patients were classified as super-responders; 33.9% (20/59) were good-excellent responders, and 22% (13/59) as poor-moderate responders.

AERD was significantly less frequent among super-responders than non-super-responders (42.3% vs. 69.7%, *p* = 0.035) (Table 3). At baseline, super-responders showed higher BEC and BNC values than non-super-responders—445 vs. 302 cells/μL and 4,170 vs. 3,600 cells/μL, respectively—while serum total IgE did not differ. No differences were observed in prior sinus surgeries (*p* = 0.945) or previous biologic use (*p* = 0.848) between the two groups. In contrast, post-treatment surgeries (*p* = 0.005) and corticosteroid dependence (*p* < 0.001) differed significantly, consistent with the absence of rescue therapy among super-responders. After 2 years of mepolizumab treatment, super-responders demonstrated significantly greater improvement, reflected by lower clinical outcome scores.

**Table 3:** Disease and clinical characteristics of CRSwNP patients: comparison of super-responders and non-super-responders to mepolizumab after 24 months of treatment.

Variables	Super-responders^a^ (*n* = 26)	Non-super-responders^b^ (*n* = 33)	*p*-value
Personal characteristics			
Sex (male)	11 (42.3%)	10 (30.3%)	0.339
Age	59.5 (45.7–70.3)	55 (47–63)	0.427
Comorbidities and allergies			
Asthma	26 (100%)	33 (100%)	
ACT score	23 (21–25)	22 (19–25)	0.523
AERD	11 (42.3%)	23 (69.7%)	0.035
Aeroallergens sensitization	13 (50%)	18 (54.5%)	0.728
Baseline biomarkers			
Blood eosinophils/μL	445 (160–635)	302 (120–475)	0.023
Serum total IgE IU/mL	69 (27.3–121.8)	127.7 (34.5–218.5)	0.047
Neutrophils/μL	4,170 (3,175–5,987.5)	3,600 (3,170–4,525)	0.016
Disease control			
Endoscopic sinus surgery	23 (88.5%)	29 (87.9%)	0.945
Post-biologic surgeries	0 (0%)	3 (9.1%)	0.005
Previous biologic therapy	2 (7.7%)	3 (9.1%)	0.848
Corticosteroid dependence	0 (0%)	14 (42.4%)	<0.001
Scores			
NPS	1 (0.8–2)	3 (1.5–4)	<0.001
Lund-Mackay staging score	22 (18–22)	22 (17–22)	0.049
SNOT-22 total score	7.5 (2–10.5)	14 (7–21)	<0.001
VAS-total	1 (0.5–2)	2 (1–4)	<0.001
VAS-smell	1 (1–4.3)	6 (4–10)	<0.001
VAS-nasal obstruction	1 (0–2)	2 (1–5)	0.039

ACT, asthma control test; AERD, aspirin and nonsteroidal anti-inflammatory drug-exacerbated respiratory disease; ESS, endoscopic sinus surgery; IgE, immunoglobulin E; NPS, nasal polyp score; SNOT-22, sinonasal outcome test. Continuous variables are presented as median and interquartile range (IQR) and absolute and relative frequency *n* (%) for qualitative data.

^a^ Super-responders are patients who meet the EPOS/EUFOREA 2023 criteria and do not require rescue treatment (neither SCS nor ESS).

^b^ Non-super-responders are patients with poor, moderate or good response according to EPOS/EUFOREA 2023 criteria.

In our analyses, we did not observe significant variations in the response to mepolizumab according to sex (*p* = 0.339), smoking habits (*p* = 0.757), aeroallergen sensitization (*p* = 0.728), or age (*p* = 0.427).

### 3.3 Baseline biomarkers predicting super-response on mepolizumab

**Figure 1:**
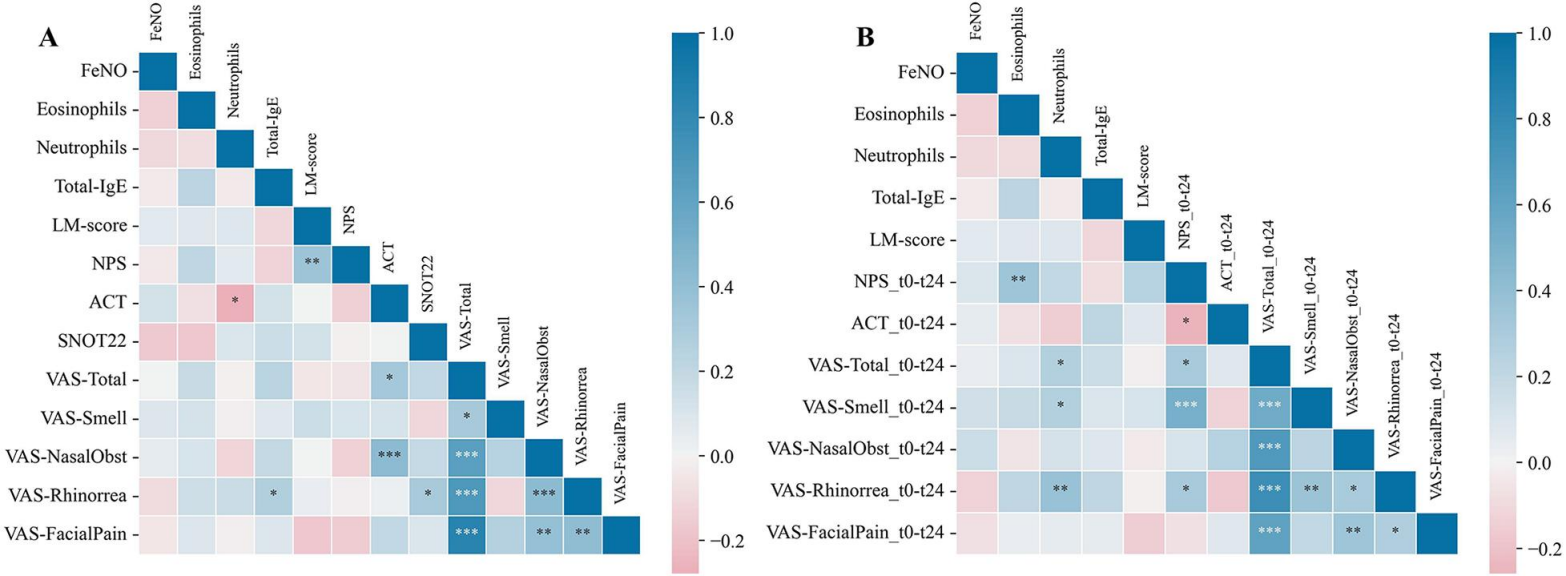
Correlation analysis of clinical outcomes with biomarkers shown as correlation matrix. **(A)** Baseline biomarkers and clinical outcomes; **(B)** baseline biomarkers and change in clinical outcomes after 2 years of mepolizumab treatment.

To investigate potential associations between baseline biomarkers (BEC, BNC, total serum IgE) and clinical outcomes in CRSwNP, correlation analyses were conducted separately for baseline outcome scores and for changes in those same outcomes at 2 years (Figure 1).

In CRSwNP (Figure 1A), baseline blood neutrophils correlated negatively with ACT score (r = −0.28, *p* = 0.033) and total serum IgE correlated positively with VAS-rhinorrhea (r = 0.27, *p* = 0.038). Over 24 months, blood neutrophils correlated with improvements in VAS-overall score (r = 0.26, *p* = 0.046), smell (r = 0.37, *p* = 0.046), and rhinorrhea (r = 0.26, *p* = 0.004), and baseline eosinophils correlated with NPS improvement (r = 0.35, *p* = 0.007) (Figure 1B).

### 3.4 Predictive performance of machine learning models

**Figure 2:**
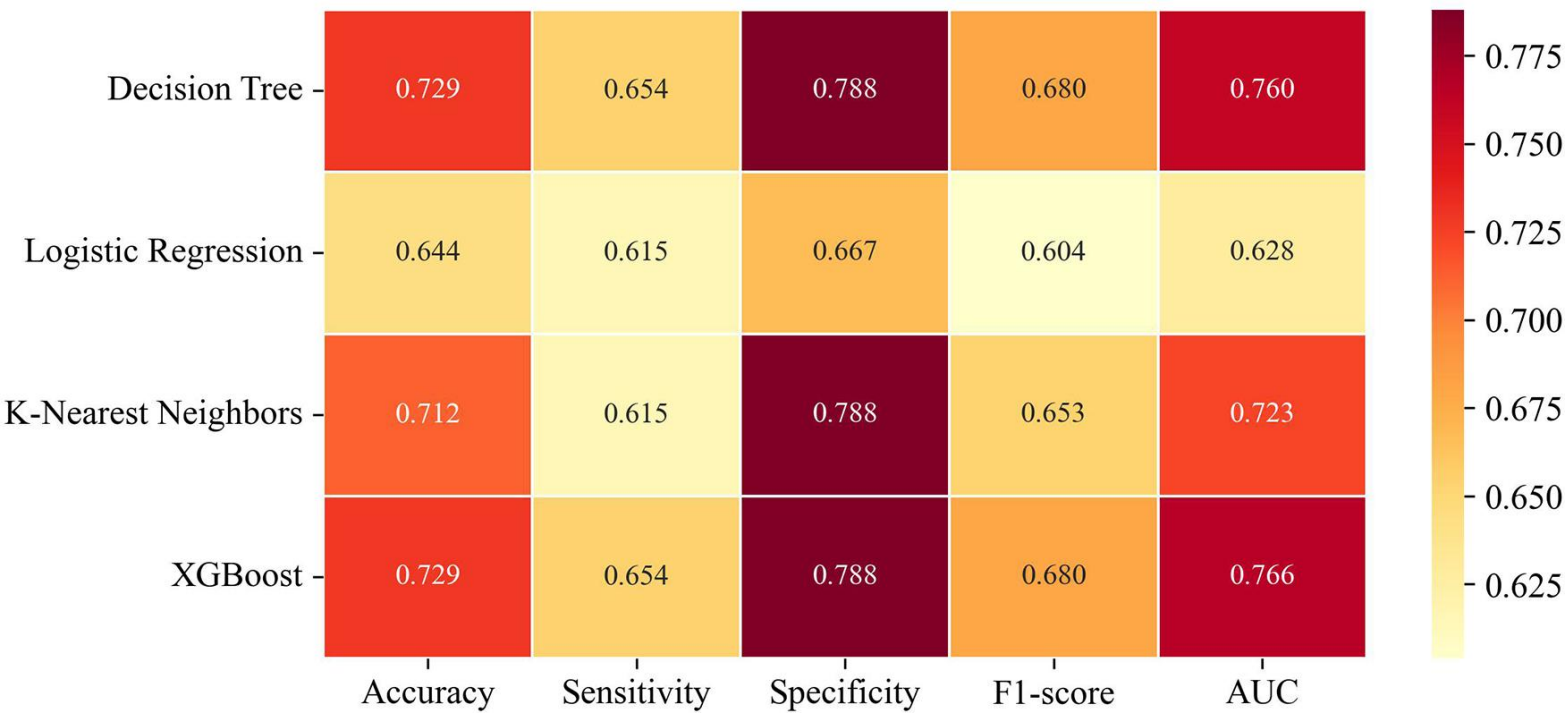
Comparative heatmap of evaluation metrics for all machine learning models.

**Figure 3:**
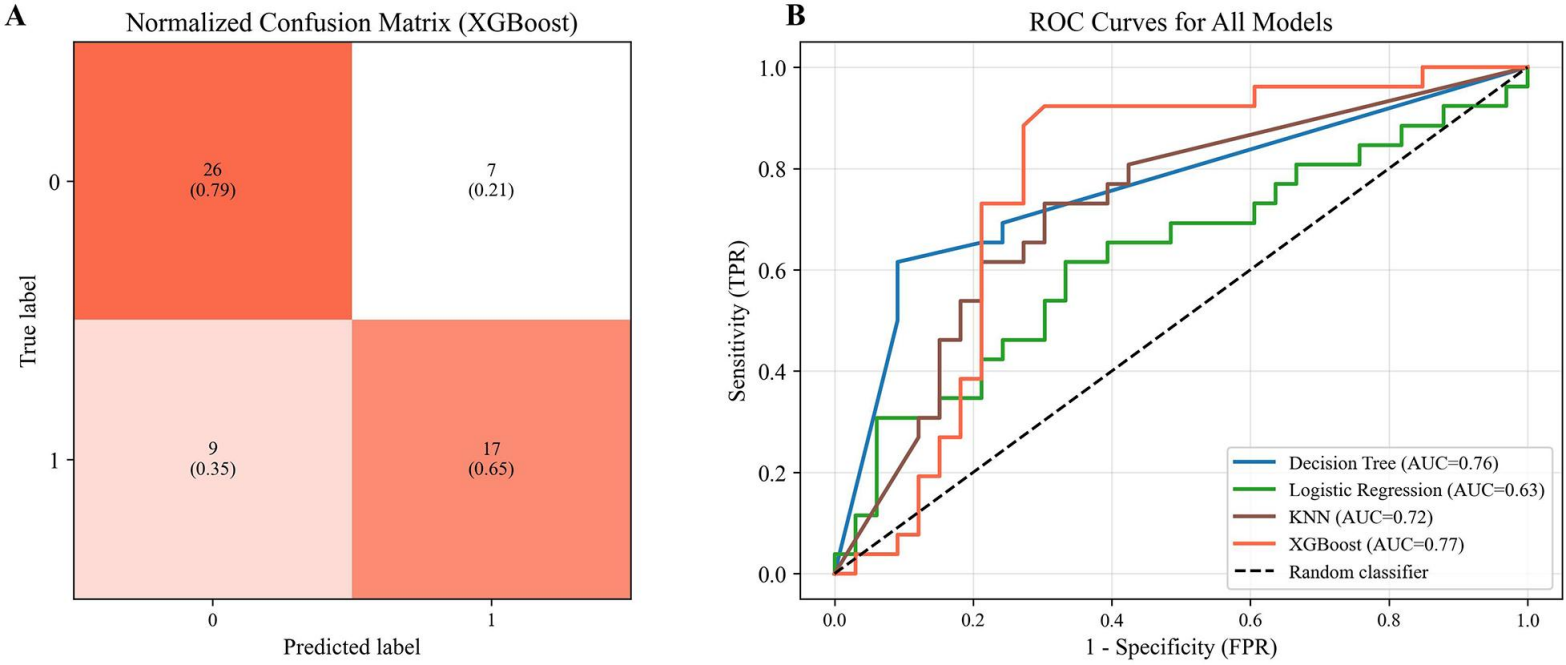
Confusion matrix of XGBoost model and ROC curves for all models. **(A)** Normalized confusion matrix of the XGBoost model illustrating classification performance. **(B)** Receiver Operating Characteristic (ROC) curves comparing the predictive performance of all evaluated models: Decision Tree (DT), Logistic Regression (LR), K-Nearest Neighbors (KNN), and XGBoost.

This study evaluated four machine-learning algorithms—DT, LG, KNN, and XGBoost—using 5-fold cross-validation under real-world conditions. Decision tree-based models (DT and XGBoost) outperformed LG and KNN, as summarized in Figure 2. XGBoost showed the highest discriminative ability (AUC = 0.766) and predicted super-response to mepolizumab at 2 years using baseline biomarkers, the Lund-Mackay score, and AERD status, achieving 72.9% accuracy, an F1-score of 0.68, 65.4% sensitivity, and 78.8% specificity, correctly classifying 43 of 59 patients (Figure 3A). Although the DT model performed similarly across most metrics, its AUC was slightly lower (0.76), as shown in the ROC comparison of all four models (Figure 3B).

### 3.5 Machine learning model interpretability

**Figure 4:**
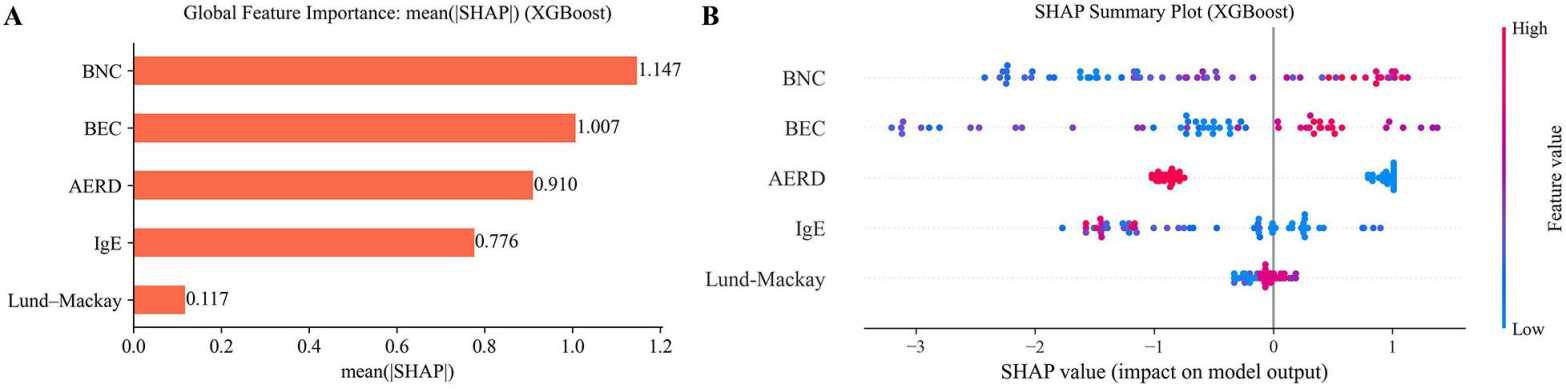
Importance and impact of top predictors of super-response to mepolizumab therapy. **(A)** bar-chart of the average SHAP value magnitude, and the mean influence of these features on the response. **(B)** SHAP summary plot in which each dot represents an individual patient, with its horizontal position indicating the magnitude and direction of the feature's impact on the model's prediction for that person. Red points correspond to higher feature values than the average and blue to low values for the variable.

Figure 4 displays the SHAP plots. Predictive factors for super-responder status were identified through SHAP analysis of the XGBoost model. Figure 4A (left) shows the mean absolute SHAP values, ranking baseline clinical features by their overall contribution to predicting treatment super-response using XGBoost. BNC, BEC and AERD status exhibited the highest mean absolute SHAP values, indicating they were the most influential features in the model's prediction of super-responder status. In contrast, IgE and Lund-Mackay score demonstrated lower mean(|SHAP|) values, reflecting a lesser impact on classification decisions.

SHAP summary plots (Figure 4B) concisely illustrate the magnitude, prevalence, and directionality of each feature's contribution to the model's predictions. Each dot represents an individual patient, positioned along the *x*-axis according to its SHAP value. The original feature value is color-coded as above (red) or below (blue) the average for that variable. Blood neutrophils and blood eosinophils, located at the top of the plot, are the most influential features in predicting a super-response to mepolizumab treatment. Higher levels of blood neutrophils and blood eosinophils (shown in red) are associated with increased SHAP values, indicating a greater likelihood of super-response. AERD status exhibited a strong discriminatory capacity as evidenced by the separation between red and blue points. AERD was consistently associated with lower SHAP values, indicating a reduced predicted probability of excellent response to treatment. This pattern is reflected in the SHAP plot, where patients with AERD (red) show a lower likelihood of being super-responders to mepolizumab compared to those without AERD (blue). Finally, IgE levels and the Lund-Mackay score had the least impact on the model's predictions and showed less consistent directional effects across patients.

**Figure 5:**
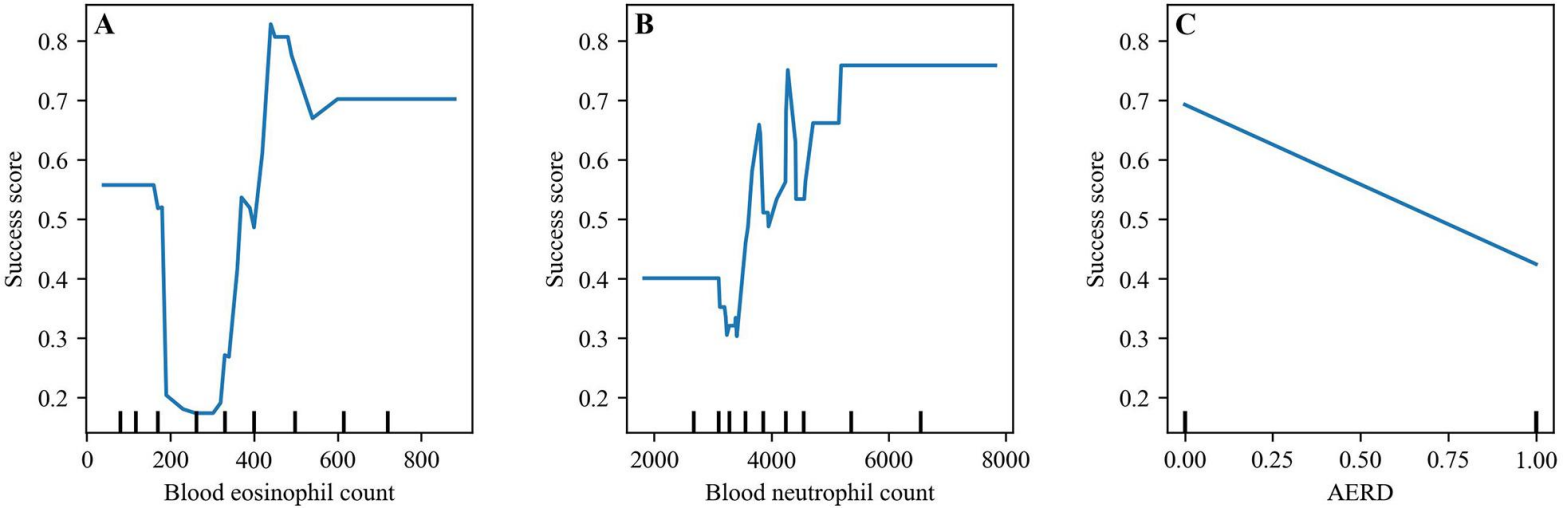
XGBoost partial dependence plots (PDP): top 3 SHAP features for super-response prediction. **(A)** Blood eosinophil count, **(B)** blood neutrophil count, **(C)** AERD.

PDPs generated using the XGBoost model illustrate the marginal effects of baseline BEC, BNC and AERD on the probability of a super-response to mepolizumab therapy. The PDP for blood eosinophils (range: 0–800) shows a non-monotonic pattern: the predicted probability initially decreases from approximately 0.6–0.2 at a count of around 150–350, followed by a sharp increase peaking near 0.8 (level around of 450), and then stabilizing around 0.7 (Figure 5A). This suggests a threshold effect where both low and high blood eosinophil counts may be associated with favorable outcomes. The PDP for blood neutrophils (range: 2,000–8,000) reveals a relatively stable probability near 0.4 up to 4,000, after which the curve fluctuates markedly, reaching values close to 0.8 before stabilizing again, indicating potential nonlinear interactions (Figure 5B). In contrast, the PDP for AERD (binary variable: 0 = non-presence, 1 = presence) displays a linear decreasing trend, with the predicted probability of super-response dropping from 0.7 to near 0.4, suggesting a significantly lower likelihood of a favorable response among AERD patients (Figure 5C).

## 4 Discussion

The effectiveness of mepolizumab in improving patients’ reported outcomes and reducing nasal polyp burden has been proven in clinical trials (11, 35) and real-world studies (7, 9, 36–41).

Due to the shared prevalence of type 2 predominant inflammation, including elevated IL-5, patients with comorbid CRSwNP and severe asthma are likely to respond well to mepolizumab (42, 43). CRSwNP, asthma and/or AERD patients have an increased disease burden because of this shared pathway (44).

To the best of our knowledge, this is the first study to use machine learning to characterize super-responders among CRSwNP patients receiving mepolizumab. For evaluating response to biologic treatment, we used adapted EUFOREA/EPOS 2023 update criteria (32). Our super-responder patients met the combined criteria with no need for either SCS or ESS. In addition, patients had to have shown improvement in clinical symptoms, comorbidities, and quality of life. After 2 years of mepolizumab treatment, 44.1% (26/59) were super-responders.

Machine learning algorithms such as DT, Random Forests (RF), Support Vector Machines (SVM), and XGBoost have demonstrated utility in guiding biologic therapy selection (45). Predictive decision tools to identify super-responders to mepolizumab therapy under defined clinical and biomarker conditions would significantly enhance the precision of treatment selection and resource allocation.

In addition to clinical variables such as AERD status and the Lund-Mackay score, our models incorporated baseline blood biomarkers (BEC, BNC, and total serum IgE) to improve prediction of super-response to biologic therapy. Among all classifiers, XGBoost achieved the highest ROC AUC. Because interpretability is essential for translating machine-learning outputs into clinically meaningful insights, we applied SHAP and PDPs to complement predictive accuracy. SHAP values provided locally consistent estimates of feature contributions, whereas PDPs offered a global view of how individual predictors influenced the model, including nonlinear and non-monotonic effects. Together, these tools enhanced model transparency and supported clinical decision-making by aligning algorithmic predictions with established pathophysiological reasoning.

SHAP summary plots show that higher levels of blood neutrophils and blood eosinophils are associated with higher SHAP values, indicating a higher likelihood of super-response. The presence of AERD in patients with CRSwNP and asthma represents a distinct endotype characterized by severe, type 2-driven inflammation and a high burden of disease (46). Recognizing AERD is essential when initiating biologic therapy, as it may influence both the magnitude and variability of treatment response. Incorporating AERD status into predictive models can enhance the precision of response stratification and support more personalized therapeutic decisions in this complex patient population. AERD status exhibited a strong discriminatory capacity. A lower SHAP value is consistently associated with its presence, indicating a lower probability of achieving an excellent response to treatment. SHAP plots show that patients with AERD are less likely to be super-responders to mepolizumab than those without.

PDPs visualize the isolated influence of individual input features on the model's predicted output by averaging predictions over all other variables. PDPs generated using the XGBoost model show marginal effects of baseline BEC, BNC and AERD on mepolizumab therapy.

In accordance with Bachert et al. (47) mepolizumab significantly reduced NPS, risk of surgery and improved nasal symptoms in CRSwNP regardless of comorbid AERD. We observed a much lower rate of AERD among super-responder patients (42.3%) compared to non-super-responders (69.7%), with a significant association between AERD status and treatment super-response (*χ*² = 4.468, *p* = 0.035). As in Baird et al. (48), patients who did not respond to biological therapy in our study were more likely to have AERD. In long-term follow-up after 2 years of treatment, AERD comorbidity might influence treatment response.

Regarding other factors predicting super-response, the Lund-Mackay score demonstrated lower mean (SHAP) values, so it has a lesser impact on classification decisions. This observation may be in accordance with Baird et al. (48) who observed no significant difference in the Lund-Mackay score between the biologic non-responders and the control group.

Several real-life observational studies (7, 9, 36–41) have demonstrated that mepolizumab improves asthma control based on a significant reduction in ACT score. Bagnasco et al. (49) showed an increase in asthma control as measured by ACT score in the first year, although values plateaued over the next 2 years. We found that mepolizumab significantly improved asthma control at 6, 12 and 24 months, with less enhancement between the 12- and 24-month periods. A significant reduction in ACT score, with an improvement of 6.5 (5.5; 8) at 2 years after mepolizumab treatment was observed. A recent real-life study (50) has also demonstrated mepolizumab long-term efficacy and safety in patients with severe asthma.

In Bagnasco's et al. (49) long-term study of mepolizumab, a higher prevalence of asthma and CRSwNP than previous studies were observed. In patients with severe asthma and CRSwNP comorbidity, mepolizumab showed long-term efficacy at 3 years. CRSwNP associated with asthma might be an indicator of good response to mepolizumab.

Our results show a significant reduction in SNOT-22 score at 6, 12 and 24 months, with greater improvements than in other real-life studies (36–41). As Garcia et al. (39) pointed out, the coexistence of asthma in all patients in our study may have impacted the poorer baseline SNOT-22 score and the larger improvement observed after 2 years. Galletti et al. (40) found that CRSwNP patients who had previously undergone ESS presented higher baseline SNOT-22 score values. In our study, 74 (88.1%) patients had previously undergone this procedure.

Real-life studies (36–41) have also demonstrated a reduction in NPS. In line with Orlando et al. (38), who found a substantial reduction (≥2 points) in NPS after 12 months of mepolizumab treatment, and significant reduction in NPS of −2.5 (−3; −2) after 24 months.

An interesting finding from our study was the VAS-smell score improvement at 6, 12 and 24 months with a significant difference between the 12- and 24-month periods of −1.5 (−2; −1). Baseline values for VAS-smell score were initially 10 (10; 10) and showed a reduction of −2.5 at 6 months. Continued improvement was noted with a reduction of −3 at 1 year; by 2 years, the reduction reached −5.5 (−6; −4). This result is in line with the *post-hoc* analysis of the SYNAPSE study (51), which found that mepolizumab improved olfaction measured by scores on the VAS-smell and SNOT-22 sense of smell/taste item.

Book et al. (52) observed no statistically significant decrease in NPS and no consistent improvement in SNOT-22 score and smell scores in the anti-IL-5 group. De Corso et al. (10) observed just slightly improved in recovery smell. Discrepancies with Book et al. may be due to the small sample size (10 patients) of his study. Patients in our study had higher total IgE levels at baseline, and 71.4% (60/84) had ≥150 blood eosinophils. As a result, they were considered to have high type 2 inflammation, in contrast with the baseline characteristics of anti-IL-5 patients in Book et al. (52).

Non-responder rates to mepolizumab of 25% were observed in SYNAPSE trial (30). According to Png et al. (53), a potential reason for high non-responder rates could be the limited efficacy of biologics in reducing NPS, although several real-life biologic efficacy studies (7, 36, 38–41) have demonstrated an overall NPS reduction of ≥2 points with mepolizumab treatment.

In accordance with Habenbacher et al. (54), we found that baseline blood eosinophil count levels were significantly positively correlated with an improvement in NPS (r = 0.35, *p* = 0.007) after 2 years of treatment.

According to Png et al. (53), the serum neutrophil count did not reach significance in predicting poor response to biologics but a trend in that direction was observed. Neither Brkic et al. (55) nor Habenbacher et al. (54) found any correlation between a high neutrophil-to-lymphocyte ratio pre-treatment and a good response to dupilumab treatment. Interestingly, in our study, super-responder patients had higher blood neutrophil baseline values compared to non-responders. BNC exhibited a positive correlation with improvement in VAS-overall symptoms score (r = 0.26, *p* = 0.046), as well as with the smell/ rhinorrhea symptom domains (r = 0.37, *p* = 0.046/r = 0.26, *p* = 0.004). BNC exhibited the highest mean absolute SHAP values, which was an important factor in predicting super-responders. According to Kratchmarov et al. (56), mepolizumab decreased neutrophil activation markers in patients with a significant positive response to IL-5 inhibition. Clinical efficacy of anti-IL-5 treatments might not be solely due to eosinophils (56), and as several patients do not respond to biologics, it is critical to identify easy biomarkers.

The EPOS/EUFOREA 2023 consensus specifies blood eosinophils over 150 cells/µL as a criterion to recommend biologic treatment (32). A non-monotonic pattern can be seen in the PDP for blood eosinophils (range: 0–800): first, the probability of super-response decreases from approximately 0.6–0.2 around 150–350, then increases sharply to 0.8 (around 450), and finally stabilizes at 0.7 for blood eosinophil values higher than 600. This may suggest a good correlation between the blood eosinophil values recommended for indicating biologic treatment and the greater probability of super-response to higher blood eosinophil values. Blood neutrophil counts have a relatively stable probability of nearly 0.4 up to 4,000. There was a linear decrease in the PDP for AERD, with the predicted probability of super-response dropping from 0.7 to near 0.4.

BNC, BEC and AERD status exhibited the highest mean absolute SHAP values, indicating they were the most influential features in the model's prediction of super-responder status.

This real-world study suggests that mepolizumab is effective in patients with severe CRSwNP and comorbid asthma, particularly those without coexisting AERD. Our study demonstrated that mepolizumab effectively decreased nasal polyp size and alleviated nasal obstruction. This led to a reduced necessity for both sinonasal surgery and the use of SCS. Mepolizumab significantly enhanced sinonasal symptoms and overall HRQoL, all while maintaining an acceptable safety profile. Higher baseline blood eosinophil levels were associated with an increased likelihood of a super-response to mepolizumab, supporting their potential role as predictive biomarkers and reinforcing the link previously observed in eosinophilic asthma (57). Clinical research on biomarkers for biologic therapies in CRSwNP remains at an early stage (58), and further studies are needed to identify those most predictive of optimal treatment response, in accordance with the EPOS/EUFOREA 2023 criteria.

There are some potential limitations to this study. The relatively small sample size and the fact that the study was conducted at a single center make it difficult to generalize our findings. These findings need to be validated in a multicenter study with a bigger sample size. The National Health System in Spain has covered mepolizumab for CRSwNP since January 2023. This means many patients have received this biologic before this date when asthma comorbidity was present. All patients in our study had asthma. There was no placebo control group, and all patients were treated with mepolizumab. Furthermore, 25 patients have not reached the 24-month follow-up period, due to differing stages of follow-up at the time of analysis, and 10 patients were not required to undergo ESS prior to mepolizumab, which may introduce a potential attrition bias. Our results should be interpreted with these considerations in mind.

## 5 Conclusions

Patients with severe CRSwNP maintained reduced NPS and improved sinonasal symptoms post-treatment, suggesting that targeted IL-5 inhibition is effective in CRSwNP.

Machine learning models, particularly XGBoost, can predict real-world super-response to mepolizumab in severe CRSwNP by identifying key predictors such as high baseline count of eosinophils, high baseline blood neutrophils, and no-AERD comorbidity.

Machine learning-based predictive modeling in our study led us to identify CRSwNP patients who showed a super-response after 2 years of mepolizumab treatment.

Machine learning can predict individual responses to different biologic therapies and guide potentially more effective options based on these preliminary results, leading to more effective CRSwNP treatment. The efficacy of this method in predicting response to biologic therapies requires larger datasets in real-world scenarios.

## Data Availability

The raw data supporting the conclusions of this article will be made available by the authors, without undue reservation.
